# Decoding Variants of Pyrite, Arsenopyrite, and Marcasite Using an Electron Counting Rule

**DOI:** 10.1002/anie.202502322

**Published:** 2025-05-05

**Authors:** Kristian Witthaut, Sandra Kreiner, Dirk Johrendt

**Affiliations:** ^1^ Department of Chemistry Ludwig‐Maximilians‐Universität München Butenandtstrasse 5–13 (D) 81377 München Germany

**Keywords:** Arsenopyrite, Crystallography, Electron counting rule, Marcasite, Pyrite, Transition metal compounds

## Abstract

Pyrite (P), marcasite (M), and arsenopyrite (A) are of significant historical and contemporary interest. Compounds adopting these structure types are studied for their crystallographic properties and potential applications in energy storage and conversion. Despite numerous investigations since the 1970s, understanding the chemical behaviour of pyrite, marcasite, and arsenopyrite remains limited. This study proposes a new *structure determining electron* (SDE) rule to systematise these compounds without relying on formal charge assignments. The SDE rule predicts structure types based on the distribution of non‐localised electrons around transition metals, providing a framework for identifying and categorising related compounds. We observe good agreement with literature data and furthermore synthesised nine new ternary compounds within the Pt/Ir‐Ge–As/Sb systems, demonstrating the applicability of SDE. Our findings reveal that these compounds can be seen as layer configurations of P, M and A, enhancing our understanding of their chemical diversity. This work not only categorises existing compounds, but also paves the way for future exploration of new materials, highlighting the structural potential of P, M, A‐related compounds beyond traditional frameworks. Preliminary results indicate that the type of layers influences physical properties, such as electrical conductivity, warranting further investigation into the relationship between structure and function in these compounds.

## Graphical abstract

A new ‘structure determining electron’ (SDE) rule allows identifying marcasite (M), arsenopyrite (A), and pyrite (P) related compounds based on the electron distribution around transition metals. It offers a framework for categorising structural variants as compounds with M, A, and P layers. Nine new ternary representatives, including the first to show both hetero‐ and homoatomic dumbbells, demonstrate enhanced chemical diversity.

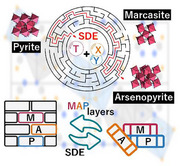

## Introduction

The compound that gives pyrite its name, FeS_2_, has been of importance since the beginning of crystallographic analysis, being among the first structures studied by L. and W. H. Bragg.^[^ ^1^ ^]^ It is already mentioned in some of the earliest scientific works from around the 1700s (e.g., ‘*pyrite aureo colore*’).^[^ ^2^, ^3^ ^]^ Hundreds of publications are published each year on the structure types and properties of pyrite (*Pa* $\bar{3}$), marcasite (*Pnnm*), and arsenopyrite (*P*2_1_/*c*). Most of these publications fall into two main categories. The first group studies investigates naturally occurring samples from a geological point of view.^[^ ^4^, ^5^, ^6^, ^7^, ^8^, ^9^ ^]^ The second group consists of ab initio DFT studies on the properties of old compounds, some of which have been known for over a hundred years.^[^ ^10^, ^11^, ^12^, ^13^, ^14^ ^]^ FeS_2_ (pyrite) is a particularly well‐studied compound due to its high mineralogical abundance and non‐toxicity. Recent publications have proposed that it may serve as a promising cathode material in Li‐batteries,^[^ ^15^, ^16^ ^]^ a thermoelectric material,^[^ ^17^ ^]^ and a single‐crystal photoelectrode,^[^ ^18^, ^19^ ^]^ as well as a potential candidate for photocatalysis,^[^ ^20^ ^]^ photocapacitor,^[^ ^21^ ^]^ photodetector,^[^ ^22^ ^]^ and has good photovoltaic performance^[^ ^23^, ^24^, ^25^, ^26^, ^27^, ^28^, ^29^ ^]^ which seems to be enhanced by pyrite–marcasite phase junctions.^[^ ^30^ ^]^

Given the considerable research effort that has been invested, it is surprising that limited progress has been made since the 1970s in understanding of the three classes, and in exploring new members. Following the discovery of new materials in the 1970s, coupled with the expanding theoretical understanding of chemical bonding, several new models have emerged to describe the aforementioned classes. However, some of these models are contradictory. In addition, the exponential growth of data has led to an overwhelming number of partially outdated publications.

In 1965, F. Hulliger and E. Mooser were among the first to provide a rationalisation of the bonding situation in pyrite, marcasite, and arsenopyrite.^[^ ^31^, ^32^ ^]^ The relationship between the structure types has been known for some time.^[^ ^33^, ^34^, ^35^ ^]^ By synthesising a large number of binary representatives, they established a correlation between the respective structure type and the d electrons of the transition metal T. Their approach was a straightforward ionic model based on crystal field theory, in which diatomic anions are given formal charges namely T^2+^X_2_^2−^, T^3+^XY^3−^, and T^4+^Y^4−^, which satisfy the ‘8−N’ rule. The reason for the different structure types was explained by a Jahn‐Teller distortion, which was also postulated to be the reason for the formation of metal‐metal bonds (T─T pairs) in the arsenopyrite structure type.

Only 5 years later G. Brostigen and A. Kjekshus build on this work and developed their ‘expansion model’.^[^ ^36^, ^37^, ^38^ ^]^ The model is based on molecular orbital (MO) considerations of pyrite and marcasite where T─T interactions are repulsive leading to an expansion of the unit cell. The advantage of this model was its more covalent approach, which has later been criticised for being inconsistent with the high charges (e.g., Fe^4+^) and the small bandgap or even metallic behaviour found for the compounds. The additional interactions required for Y_2_^4−^ (e.g., As_2_^4−^) pairs were rejected, as was the idea that π bonds play an important role. It was expected that compounds of the arsenopyrite type would only occur for d^5^ configurations of the transition metal.

In 1972, J. B. Goodenough argued that the T─T interactions for half‐filled orbitals cannot be repulsive by nature, but rather must be attractive. He subsequently explained the phenomenon of the elongation along the *c*‐axis as a consequence of repulsive T–X interactions, and postulated that the compounds are dominated by T–X interactions rather than T─T interactions.^[^ ^39^ ^]^ However, he still assumed different formal oxidation states of iron for each of the three structure types. In 1981, J. A. Tossell et al. conducted quantitative MO calculations that strengthened a T–X–T interaction hypothesis over the T‐T interaction hypothesis. However, their model still relied on the X_2_ groups behaving as 14 or 12 valence electron systems.^[^ ^40^ ^]^

With advances in technology over the next years evidence was presented from Mössbauer spectroscopy that all three minerals appear to adopt the oxidation state of Fe^2+^, challenging the assumptions of the previous models.^[^ ^41^ ^]^ This was supported by more recent studies. An X‐ray photoelectron spectroscopy (XPS) analysis of the oxidation state of Fe in FeAs_2_ observed evidence for the same oxidation state in pyrite, marcasite, and arsenopyrite.^[^ ^42^ ^]^ A fundamental finding was presented in 2014 with the analysis of experimental and theoretical electron density of FeS_2_. It indicates that the atomic charge appears to be significantly smaller than that of Fe^2+^ and S^1^.^[^ ^23^ ^]^ There authors assigned this to covalent Fe–S contributions.

Considering this current state of research, we propose a ‘minimal bonding model’ with a straightforward ‘structure determining electron’ (SDE) rule that operates without the assignment of formal charges. The aim of this study is not to provide an exhaustive explanation, but rather to facilitate a comprehensive and effective systematisation of existing compounds, as well as to predict new representatives and combinations of the structural types of pyrite, marcasite, and arsenopyrite. As additional proof of concept, we present nine new compounds to expand the data about structure types and their understanding. These adopt either the known structure types of marcasite (M), arsenopyrite (A) and pyrite (P) or stacking variations. Some of these stacking variations are the first examples for M/A/P related structures to show homo‐ and heteroatomic dumbbells to exist within the same compound.

## Results and Discussion

It is established in the literature that these structure types are closely related, although the precise nature of the relationship and the bonding situation remains unclear.^[^ ^34^, ^35^, ^43^ ^]^ We begin by introducing the three ‘basic’ structure types and elucidating their structural relationship. For this, we use the marcasite structure type to include topologically similar structures that just differ by the degree of distortion but conserve the space group *Pnnm*. We also neglect the polymorphism of some of the compounds as it is already described in the literature. The most prominent example for this is FeS_2_, as it is the composition of both minerals pyrite and marcasite.^[^ ^23^, ^44^ ^]^ We then highlight the difficulty in comprehending the bonding situation and furthermore we introduce a novel electron‐counting rule based on a ‘minimal bonding model’, which appears to systematise the structure types effectively.

Subsequently, the newly discovered layered structure variations will be presented. In order to facilitate comprehension, we have devised a nomenclature that derives the structure from combinations of the arsenopyrite (A), pyrite (P), and marcasite (M) structure types.

**Figure 1:**
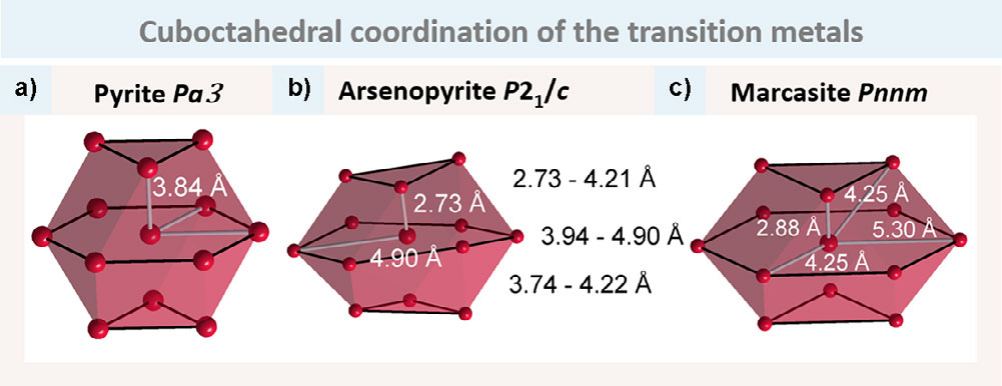
Illustration of the cuboctahedral coordination of T in the three structure types a) pyrite, b) arsenopyrite, and c) marcasite. The distances are only for an easy comparability, and thus rounded to two digits. For arsenopyrite, each distance is different, therefore ranges are given in addition to the longest, and shortest distance shown in white.

The compounds representing the ‘basic’ structure types are FeS_2_ (*Pa* $\bar{3}$, pyrite),^[^ ^45^ ^]^ FeAsS (*P*2_1_/*c*, arsenopyrite)^[^ ^46^ ^]^ and FeAs_2_ (*Pnnm*, marcasite).^[^ ^47^ ^]^ All three can be regarded as TX_2_ structures, where T represents a transition metal (Fe, Pt, Ir, …) and X denotes a main group element (S, As, Ge, …). An initial insight into the structural relationship between T and its neighbouring atoms can be gained from an examination of the packing of T, as illustrated in Figure 1. In pyrite (Figure 1a), the T atoms form an undistorted TT_12_ cuboctahedron (ellipsoid shape = 0.000, analysed with PIEFACE^[^ ^48^ ^]^) with all neighbouring T atoms being equidistant from each other. This is evident from the cubic space group *Pa* $\bar{3}$. In marcasite (Figure 1c) this cuboctahedron is strongly compressed along its diagonal, resulting in two short T‐T distances of 2.88 Å and two long T─T distances of 5.30 Å (ellipsoid shape = 0.051). The remaining eight T atoms, which are equidistant from another, form a tetragonal coordination. Arsenopyrite (Figure 1b), exhibiting the lowest symmetry, displays the most pronounced distortion, with all neighbouring T atoms exhibiting distances that vary between 2.73 and 4.90Å (ellipsoid shape = −0.049).

**Figure 2:**
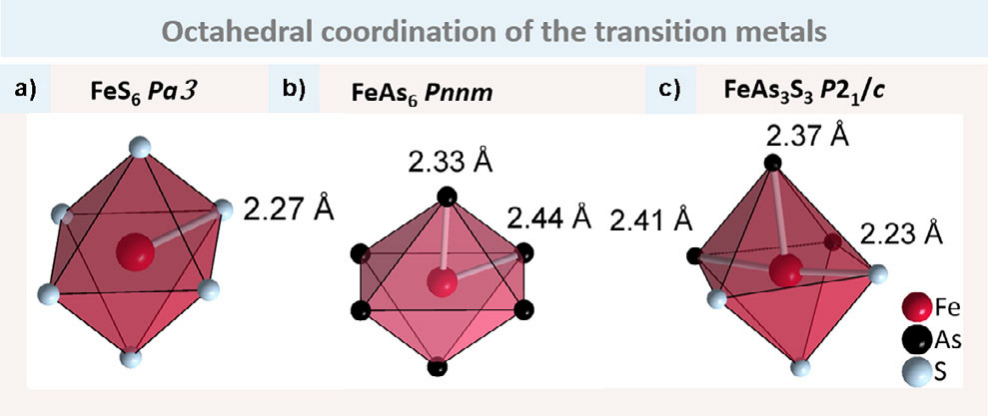
Octahedral coordination of T by X in the examples of a) FeS_6_ in pyrite structure type, b) FeAs6 in marcasite structure type and c) FeAs_3_S_3_ in arsenopyrite structure type. White bonds show the distances (rounded to two digits) found in the three structure types to highlight the difference.

The polyhedra around T and X, respectively, are identical for all three structure types, although they exhibit varying degrees of distortion. The X atoms are consistently tetrahedrally coordinated by three T and one additional X. In this study, we will focus on the T–X coordination with only TX_6_ octahedra present in all compounds, as illustrated in Figure 2.

In FeS_2_ (pyrite, Figure 2a) all distances are equivalent with 2.27 Å. For FeAs_2_ (marcasite, Figure 2b) the octahedron is compressed leading to two shorter distances with 2.33 Å and four longer distances of 2.44 Å. It is evident that FeAsS (arsenopyrite, Figure 2c) exhibits the greatest degree of distortion, with Fe–S distances of approximately 2.23 Å and Fe–As distances of 2.37–2.41Å. The octahedron in FeAsS is always composed of three As and three S atoms.

At this juncture, it becomes evident that the distances are relatively similar. In a subsequent section, the rationale behind the distortion is discussed in depth, and it will be observed that the primary factor is the disparity in the bonding angles. The connectivity of the octahedra, in terms of the number of octahedra that are interconnected, is identical for all three structure types, despite the differing realisation.

**Figure 3:**
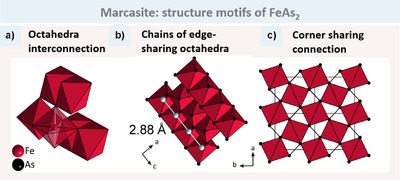
Structure motifs of FeAs2 as an example of the marcasite structure type. a) The Figure depicts the octahedral interconnection. The two apical atoms are only corner‐sharing with two other octahedra (highlighted in white), while the four equatorial atoms are each corner‐ and edge‐sharing with two other octahedra. b) Chains of edge‐sharing octahedra along the *c* axis, with the shortest Fe─Fe distance (2.88 Å) highlighted in bright bonds. c) The connection between the octahedra‐chains in the *a‐b* plane is only via octahedra corners. As─As bonds are shown in black.

For the sake of convenience, we will begin with the FeAs_2_ (marcasite) structure, as shown in Figure 3, for which only one type of octahedron is present. In all octahedra, each corner connects with two other octahedra (Figure 3a). This allows us to differentiate between two types of connections. Within the octahedron, we have two opposite X atoms that are only corner‐ sharing. This direction is defined as the apical connection (brightly coloured bonds). The other X atoms form the equatorial connection. These atoms connect to one neighbouring octahedron edge‐sharing and one corner‐sharing. In the marcasite structure type, the T atoms along the edge‐sharing direction are all equidistant, forming infinite chains (Figure 3b). In addition to the octahedral connection, a further X─X bond is formed between two X atoms (Figure 3c).

**Figure 4:**
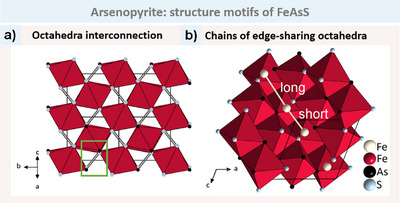
a) The octahedral‐chains in FeAsS (arsenopyrite) exhibit a similar connection to that observed in marcasite. Only As─S bonds are formed and shown in grey (green box). b) The chain of edge‐sharing octahedra is oriented along the [101] direction. In arsenopyrite, the octahedra form pairs along the chains, resulting in alternating long and short Fe─Fe (T─T) distances (bright bonds). The shorter distances are more likely to be a consequence of the octahedral geometry than to represent actual Fe─Fe bonds.

We now turn our attention to the FeAsS (arsenopyrite) structure. As previously stated, the connectivity for arsenopyrite is identical to that of marcasite. In fact, these two structures are highly analogous, as revealed by the direct comparison of Figures 3c and 4a. They differ only in the degree of deformation, which reduces *Pnnm* to *P*2_1_/*c*. The octahedra are once again linked by edge‐sharing chains along the equatorial plane and corner‐sharing connections along the apical direction. Figure 4a illustrates the corner‐sharing connection, whereas the edge‐sharing connection extends downwards into the Figure. The most notable distinction between this structure type and the previously discussed marcasite structure type is that the T atoms along the chains no longer exhibit an evenly spaced arrangement but instead alternate between short and long distances (see Figure 4b). In older publications, this led to the assumption that T─T bonds were the reason for the T_2_ pairs instead of T_∞_ chains. However, newer investigations have not found any indications for T─T bonds, and instead, the alternating T‐T distances could be attributed to the TX_6_ octahedral geometry. This hypothesis will be justified in a later section.

**Figure 5:**
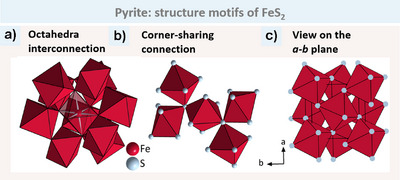
a) The interconnection of the TX_6_ octahedra in pyrite occurs exclusively at the corners. However, as in the cases of arsenopyrite and marcasite, the connection is always established with two other octahedra. b) The rotation of the octahedra out of the ‘edge‐sharing plane’ leads to corner connectivity only, but conserves the coordination number. c) An overview of the *a‐b* plane of the pyrite structure type is presented for the example FeS_2_. Because of the corner connection, no chains of edge‐sharing octahedra are formed.

Prior to undertaking a detailed analysis, it is first necessary to provide an overview of the pyrite structure type. Figure 5 illustrates the FeS_2_ as an example for the pyrite structure. As in arsenopyrite and marcasite, each octahedron corner is connected to two other octahedra. In contrast to the edge‐sharing, the octahedra in pyrite are only corner‐sharing (Figure 5a). This is possible by rotating the neighbouring octahedra out of the ‘edge‐sharing‐plane’ (Figure 5b).

Without anticipating the explanation, this seems intuitive in a geometrical way. When considering the T‐T_12_ coordination, we expect a cuboctahedron for equal‐sized ‘spherical’ octahedra. However, if we now have a deformed octahedron, our cuboctahedron starts to ‘collapse’, which is what forms the octahedral chains. In the case of pyrite, the octahedron is close to perfect symmetric. Consequently, the structure forms only corner connections.

It is curious to note that seemingly similar compounds can form different structure types. For instance, FeS₂ forms pyrite, whereas FeAs₂ forms marcasite. One might hypothesise that FeAsS forms the arsenopyrite type, by occupying a position somewhere between pyrite and marcasite. This leads to the conclusion that octahedra with different coordinating atoms must be deformed, and thus the arsenopyrite type is a ternary variant. Nevertheless, IrAs₂, for example, forms the arsenopyrite type. In contrast, IrGeAs, a new compound presented later in this paper, forming the marcasite type. It is also evident that merely counting valence electrons (VE) is insufficient. Table 1 provides an overview of some representatives with their respective structure type and number of valence electrons.

**Table 1:** The Comparison of different compounds forming the pyrite, arsenopyrite, or marcasite structure shows that the structure type (ST) is not an obvious consequence of the respective composition or the compounds having a fixed number of valence electrons (VE).

ST	VE	ST	VE	ST	VE
Pyrite	20	Arsenopyrite	19	Marcasite	18
FeS_2_		FeAsS		FeAs_2_	
PtAs_2_		PtGeAs		PtGe_2_	
IrAsS		IrAs_2_		IrGeAs	
NiAsSe	21			CrSb_2_	16
MnS_2_	19				

Previous attempts to systematise the structure types have been unsuccessful due to limited data. Consequently, previously valid descriptions have been shown to be flawed with the discovery of new compounds. One of the earliest models is the ‘expansion model’.^[^ ^36^, ^37^, ^38^ ^]^ The fundamental concept is that main group elements in these compounds must have complete octets. Since, they assume compounds forming the marcasite or arsenopyrite structure type to form T─T bonds, this leads to a division of the structure types according to the d‐orbital configuration of the transition metal. Without delving into the specifics, it becomes evident that this model is inadequate for compounds comprising main group elements with an atomic number less than 5. In the case of PtGe_2_, for instance, a Ge_2_ pair requires six electrons to fill its octet, which would result in the formation of Pt^6+^.

As the number of compounds continued to grow, new models were proposed. One such model builds upon the initial model, suggesting that a fixed number of electrons is responsible for the formation of the respective structure type.^[^ ^40^ ^]^ If the X_2_ main group elements have 12 VE, the marcasite structure is preferred. Conversely, if they have 14 VE, the pyrite structure is preferred. The deeper analysis of compounds such as PtAs_2_ (pyrite) and FeAs_2_ (marcasite) has already led to difficulties. In order for this hypothesis to be valid, the compounds in question would have to be Fe^2+^As_2_^2−^ with As‐As double bonds and Pt^4+^As_2_^4−^ with As‐As single bonds. However, both compounds exhibit similar As‐As bond length of 2.41 Å. All of these models have in common that they apply expected formal charges to the elements and fulfil the octet rule. This appears to be an inadequate approach.

As previously stated, the differences in the structures are reflected in the deformation of the T‐T_12_ cuboctahedra. Our hypothesis is that this phenomenon is a consequence of the deformation of the TX_6_ octahedra and not the formation of T─T bonds.

### Minimal Bonding Model

In order to understand the octahedral deformation, it is necessary to examine the bonding situation in the TX_2_ compounds. To this end, two assumptions are made. Firstly, it is assumed that only single bonds are formed. This agrees well with the small formal charges found in the XPS findings from Schmøkel et al.^[^ ^23^ ^]^ Since all X atoms form X‐T_3_ tetrahedra, four electrons are required for the single bonds. The second assumption is based on the splitting of the d‐orbitals into t_2g_ and e_g_. The transition metal will first occupy its t_2g_ orbitals, with up to the maximum of six electrons. The electrons occupying the e_g_ orbitals together with the p electrons of X will define how much the octahedron is deformed and which structure type is adopted.

Before elucidating the consequences, it is beneficial to examine the outcome. The assumptions permit the classification of the known compounds into their respective structure types. For this, we employ the following electron‐counting rule, wherein the SDE determine the preferred structure type. To include ternary compounds, we define TX_2_ to be TXY.

$$\begin{array}{cc} \text{SDE} & =T_{\mathrm{VE}}-T_{t2g}+X_{\mathrm{VE}}-4_{\sigma }^{X}+Y_{\mathrm{VE}}-4_{\sigma }^{Y}\\ & =T_{\mathrm{VE}}-T_{t2g}+\left(X_{\mathrm{VE}}+Y_{\mathrm{VE}}\right)-8_{\sigma } \end{array}$$

T_VE_ is the number of VE of the transition metal. T_t2g_ is the number of electrons in the t_2g_ d orbitals without the s electrons, with a maximum of six. X_VE_ and Y_VE_ are the number of VE of the main group element X and Y, respectively. For both main group elements four electrons are subtracted for the single bonds (4^X^_σ_ and 4^Y^_σ_). This can be further simplified for the here investigated structures. The T atoms have a configuration of s^2^t_2g_^a^e_g_^b^ and the X/Y atoms a s^2^p^c^ configuration. Thus leaving out the s electrons we can count T_eg_, the number of electrons in the e_g_ orbital, because T_VE_ – T_t2g_ – T_s2_ = T_eg_. Consequently we only count the p electrons of the X/Y atoms. Where Ep is the number of p electrons from X (X_p_) and Y (Y_p_). As we now count 6 s‐electrons less we subtract 2 instead of 8:

$$\begin{array}{cc} \text{SDE} & =T_{\mathrm{eg}}+X_{p}+Y_{p}-2\\ & =T_{\mathrm{eg}}+\text{Ep}-2 \end{array}$$

The number of SDE then allows a classification according to:


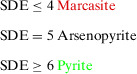




Exemplary calculations are shown in the next section. Table 2 presents a matrix of different TXY combinations. In order to limit the scope of the analysis, only transition metals from the 4^th^ to the 10^th^ transition metal group (T) are included. Furthermore, the first three main groups for the main group elements X and Y are excluded. The matrix is coloured according to the predicted structure type, as determined by the SDE rule. The marcasite structure type is highlighted in red, the arsenopyrite structure type in orange and the pyrite structure type in green. Since some of the ‘pyrite’ compounds (e.g., CoAsS) contain heteroatomic dumbbells, they cannot conserve the cubic space group. The description as P remains suitable because the adopted structures can be derived from group‐subgroup relations. The same applies for structures derived from arsenopyrite and marcasite.

**Table 2:** Overall good agreement with the SDE rule visualised by combinations of transition metal T (number of d electrons from 4 to 10) with main group elements X and Y in the groups 4 to 6. The predicted structure types, according to their SDE, are highlighted in red for marcasite, orange for arsenopyrite and green for pyrite. Existing examples from the ICSD Database^[^ ^49^, ^50^ ^]^ and our newly found compounds are given as examples for specific combinations.

		T (d electrons)						
X	Y	4	5	6	7	8	9	10
4	4					PtGe_2_		
4	5				IrGeAs	PtGeAs		
4	6					PtSnS		
5	5	CrSb_2_		FeAs_2_	RhBi_2_	PtAs_2_	AuSb_2_	
5	6			FeAsS	CoAsS	NiAsSe		
6	6		MnS_2_	FeS_2_	CoS_2_	NiSe_2_	CuS_2_	ZnS_2_

If at least one compound exists in the ICSD Database,^[^ ^49^, ^50^ ^]^ the respective box is marked by an example. A more detailed analysis is provided in Table S4, were 68 TX_2_ compounds from the ICSD Database exhibit a 95.6% agreement with the SDE rule.

The SDE rule appears to be an effective predictor for the distinction of the Marcasite, pyrite, and arsenopyrite structure types, with so far just a single exception. Some of the compounds can be transformed into marcasite through the application of sufficient pressure, while others have been investigated solely as minerals with impurities, which cannot be considered.^[^ ^37^ ^]^ The only notable exception occurs when the size difference between transition metals and main group elements becomes too pronounced. For instance, NiSb_2_ forms the marcasite structure type instead of pyrite, as predicted by the SDE rule. This can be explained by considering the electron distribution surrounding the transition metal. If the structure types are a result of the electron distribution, then this is partially size dependent. If the transition metal is very small compared to the main group element, the octahedral void will be sufficiently large for the transition metal to fit in, even with higher electron numbers, and the main group elements are pushed together.

What can be gleaned from the numerical data? A ‘symmetric’ octahedron with nearly equal bonding distances and angles is observed when all corners are equal. This indicates the need for six T–X bonds, which require 12 electrons. Our initial assumption is that the X atoms contribute at least six electrons to each octahedron. The SDE provides insight into the additional electrons contributed by T and X. With SDE = 6, we have 12 electrons and thus a ‘symmetric’ octahedron with an almost spherical distribution of the electron density. This results in the formation of the ‘perfect’ cuboctahedron. However, when the number of electrons is decreased, a non‐spherical electron distribution is enforced. The different levels of deformation become evident when examining the X–T–X bonding angles within the equatorial plane.

**Figure 6:**
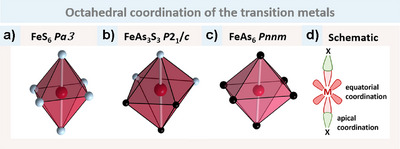
Visualisation of the definition of the apical and equatorial direction for the three structure types a) pyrite (FeS_2_), b) arsenopyrite (FeAsS) and c) marcasite (FeAs_2_) with bright bonds. d) provides a schematic view of the coordination.

The previously outlined definition of directions is still used here. The directions shared only by corners are apical and the directions shared by corners and edges are equatorial (Figure 6). As all octahedra corners in pyrite are corner‐sharing, an arbitrary direction is defined. This is justified by the crystallographic equivalence of all corners. The brightly coloured bonds highlight the apical position.

**Figure 7:**
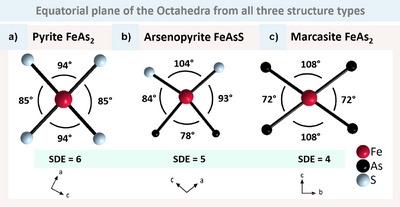
Deformation of the equatorial plane observed in the respective octahedra. a) Shows FeS_2_ as a representative example of pyrite with SDE = 6 and X‐T‐X angles close to 90°. b) Illustrates FeAsS as representative example of arsenopyrite (SDE = 5) with one large angle exceeding 100° and one small angle below 80°. c) Shows FeAs_2_, as a representative of marcasite, displaying SDE = 4 with two large angles exceeding 100° and two small angles below 80°.

In the case of SDE = 6, we observe near to 90° angles. In the case of SDE = 5, one X atom is no longer fixed to its position. This results in a single large angle of approximately 100° and the resulting octahedron is strongly distorted. Further removal of an electron (SDE = 4) leads to another angle exceeding 100° and allows for a more symmetrical distortion by compression in one direction (Figure 7).

### New ‘MAP’ Compounds

**Figure 8:**
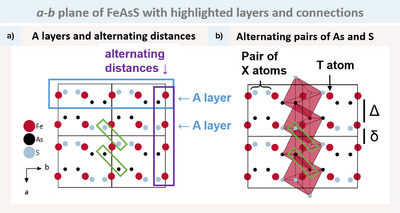
Visualisation of the *a‐b* plane of FeAsS a) One A layer of arsenopyrite octahedra is highlighted with a blue box. The edge‐sharing octahedra with alternating Fe─Fe distances run along the *a* axis highlighted with a violet box. The green box in both a) and b) shows the atoms, which are part of the edge‐sharing octahedra sides. b) Illustrates how the alternating pairs of As in black and S in grey and one red Fe atom belong to one octahedron within one layer. The long (*Δ*) and short (*δ*) Fe─Fe distances are emphasised.

The structure models presented are extracted from single crystal X‐ray diffraction data. The synthesis route is described in the supportive information and the starting materials are given in Table S1. An overview of the crystallographic data for all compounds is given in Table S3. Energy dispersive X‐ray (EDX) spectroscopy was performed to confirm the compositions and the collected data are given in Table S2. The assignment of Ge and As is supported by density functional theory (DFT) calculations, due to their low X‐ray contrast. The compounds will be introduced as layered combinations of marcasite (M), arsenopyrite (A) and pyrite (P). The *a‐b* plane of all new compounds provide comprehensive information in a single figure. Figure 8 exemplifies this for FeAsS.

Along the *b* direction (highlighted by the blue box), a single layer can be seen. All new compounds show this structure motif with alternating pairs of X atoms separated by T atoms (see Figure 8b). The octahedron around Fe in Figure 8b shows how the pairs of As and S belong to the middle Fe atom. This is enough information to use the SDE rule to identify the type of octahedra in each layer in question and thus identify the layer itself as M, A or P. For example, here in the blue box, we see alternating pairs of bright S atoms and black As atoms separated by red Fe atoms.

$$\begin{array}{cc} \text{SDE} & =T_{\mathrm{eg}}+X_{p}+Y_{p}-2\\ & ={\text{Fe}}_{\mathrm{eg}}+S_{p}+{\text{As}}_{p}-2\\ & =0+4+3-2=5 \end{array}$$

Fe_eg_ are the electrons of Fe e_g_ orbitals. S_p_ and As_p_ are the four and three p orbital electrons of S and As, respectively. The calculation gives us SDE = 5.We therefore call that layer an A layer (arsenopyrite). Looking at the subsequent layer, it also exhibits alternating As and S, yet in a reversed configuration. Again using the SDE rule we identify this layer to be an A layer, since SDE = 5. This means our structure exhibits solely arsenopyrite layers, as we already knew it would.

This may need some additional justification, because by looking at the pairs of X atoms in the blue box, we are only looking at two seemingly arbitrary edges of an octahedron. However, the occupation of all octahedra is always such that either all atoms are the same or two opposite sides are different (see Figure 6). This means looking at two opposite edges only is enough to know what kind of octahedra is formed around the specific central T atom. In reality, the layers are therefore not independent. This becomes clear when examining along the *a* direction.

Figure 8b shows how the octahedra connect. For FeAsS we see the edge‐sharing, as described in a previous section. The green box highlights the edge‐sharing atoms, which are always of the same type, As or S. In the case of FeAsS this has the consequence, that the first layer of arsenopyrite can only be followed by either an inverted layer of arsenopyrite (which is the case) or by a layer of pyrite (FeS_2_) and not by a layer of FeAs_2_. Fortunately, this is only important when thinking of new combinations, not when identifying the structure, because we name each layer separately.

Another advantage of this representation is that we see the influence of the layers on the T─T distances (violet box, Figure 8a) that follow from the stacking. For FeAsS we already saw that we have alternating long (*Δ*) and short (*δ*) distances. Given that both the new compounds, PtGeAs and PtGeSb, crystallise in the arsenopyrite structure type (*P*2_1_/*c*), we expected the same picture as described for FeAsS.

**Figure 9:**
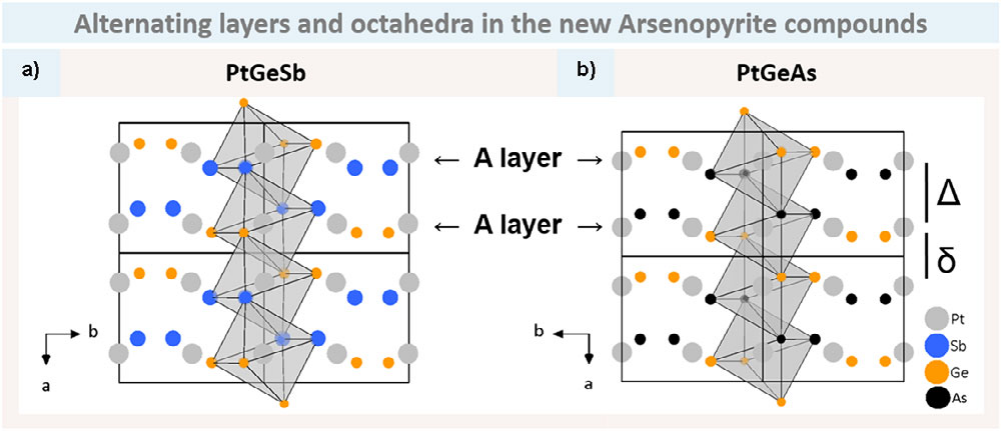
*a‐b* plane with highlighted octahedra chains of a) PtGeSb, with Pt in grey, Ge in orange and Sb in blue; b) PtGeAs, with As in black. The A layers run along the *a* axis and the alternating Pt–Pt distances are emphasised by Δ (long) and δ (short) for PtGeAs. (Space group *P*2_1_/*c*).

For PtGeSb (Figure 9a), alternating pairs of Ge and Sb are observed along the *b* direction, while for PtGeAs (Figure 9b), alternating pairs of Ge and As are observed. Both layers are consistent with SDE = 5, and therefore can be identified to be A layers of arsenopyrite.

**Figure 10:**
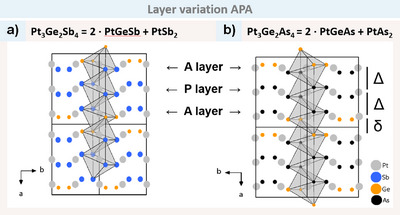
The *a‐b* plane, with the octahedra chains highlighted in a) Pt_3_Ge_2_Sb_4_, where Pt is in grey, Ge in orange and Sb in blue, and b) Pt_3_Ge_2_As_4_, with As in black. The A and P layers are pointed out for the first unit cell. The long (*Δ*) and short (*δ*) distances are emphasised for Pt_3_Ge_2_As_4_. (Space group *P*2_1_/*c*).

The practicality of the description is evident in the subsequent new compounds, Pt_3_Ge_2_Sb_4_ and Pt_3_Ge_2_As_4_ (both space group *P*2_1_/*c*). To illustrate, taking Pt_3_Ge_2_Sb_4_ as an example, we observe alternating Sb and Ge pairs as found in PtGeSb, which result in an arsenopyrite layer (Figure 10a). The subsequent layer is comprised solely of Sb pairs. This is a Pyrite layer, as observed in PtSb_2_ (pyrite) and consistent with the SDE rule (SDE = 6).

$$\begin{array}{cc} \text{SDE} & =T_{\mathrm{eg}}+X_{p}+Y_{p}-2\\ & ={\text{Pt}}_{\mathrm{eg}}+{\text{Sb}}_{p}+{\text{Sb}}_{p}-2\\ & =2+3+3-2=6 \end{array}$$

The last layer is again comprised of alternating pairs of Ge and Sb, and thus an A layer. The sum formula of Pt_3_Ge_2_Sb_4_ results from this combination of A and P layers according to 2 · PtGeSb + PtSb_2_. This indicates that the structure contains pyrite‐type octahedra in between arsenopyrite‐type octahedra. While edge‐shared octahedra chains are still observed, the distances between Pt–Pt are no longer alternating, showing short distances between the two arsenopyrite layers and long distances between the arsenopyrite and pyrite layers. This results in long (*Δ*), long (*Δ*), short (*δ*) distances along the chains as highlighted in Figure 10b. The analogous case is that of Pt_3_Ge_2_As_4_, which may be expressed as 2 · PtGeAs + PtAs_2_. This first structure variation type is thus designated as APA (A = arsenopyrite and P = pyrite). It is the first example for MAP related structures to show homoatomic (e.g., Sb–Sb) and heteroatomic (e.g., Ge–Sb) bonds in one compound.

**Figure 11:**
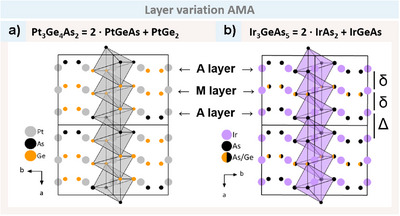
*a‐b* plane with highlighted octahedra chains along the a direction. a) Pt_3_Ge_4_As_2_ with Pt in grey, Ge in orange and As in black. Within a single unit cell, a marcasite (Pt–Ge) layer is sandwiched between two arsenopyrite (Pt–Ge–As) layers, as pointed out arrows. b) Shows the same structure for Ir_3_GeAs_5_ with Ir in violet and a mixed occupancy of Ge/As (orange/black) in the marcasite layer. The short (*δ*) and long (*Δ*) distances are emphasised for b).

In the system of Pt–Ge–As and Ir–Ge–As we were able to synthesise this variation with a marcasite instead of a pyrite layer, both shown in Figure 11. For Pt_3_Ge_4_As_2_, the middle marcasite layer is PtGe_2_, which is a known compound. The sum formula adds up to 2 · PtGeAs + PtGe_2_, and the structure type variation corresponds to AMA in the space group *P*2_1_/*c* (Figure 11a).

In contrast to the longer distances that result from the introduction of a pyrite layer, the Pt–Pt distance between marcasite and arsenopyrite layers is now shorter. This leads to the formation of short, short, long distances along the chains. Ir_3_GeAs_5_ also forms the structure variation AMA in the space group *P*2_1_/*c*, which can readily be understood using the SDE rule for each layer. The composition of layers is as follows: an IrAs_2_ (SDE = 5) arsenopyrite layer, a marcasite layer comprising IrGeAs (SDE = 4) and finally another IrAs_2_ layer (Figure 11b).

**Figure 12:**
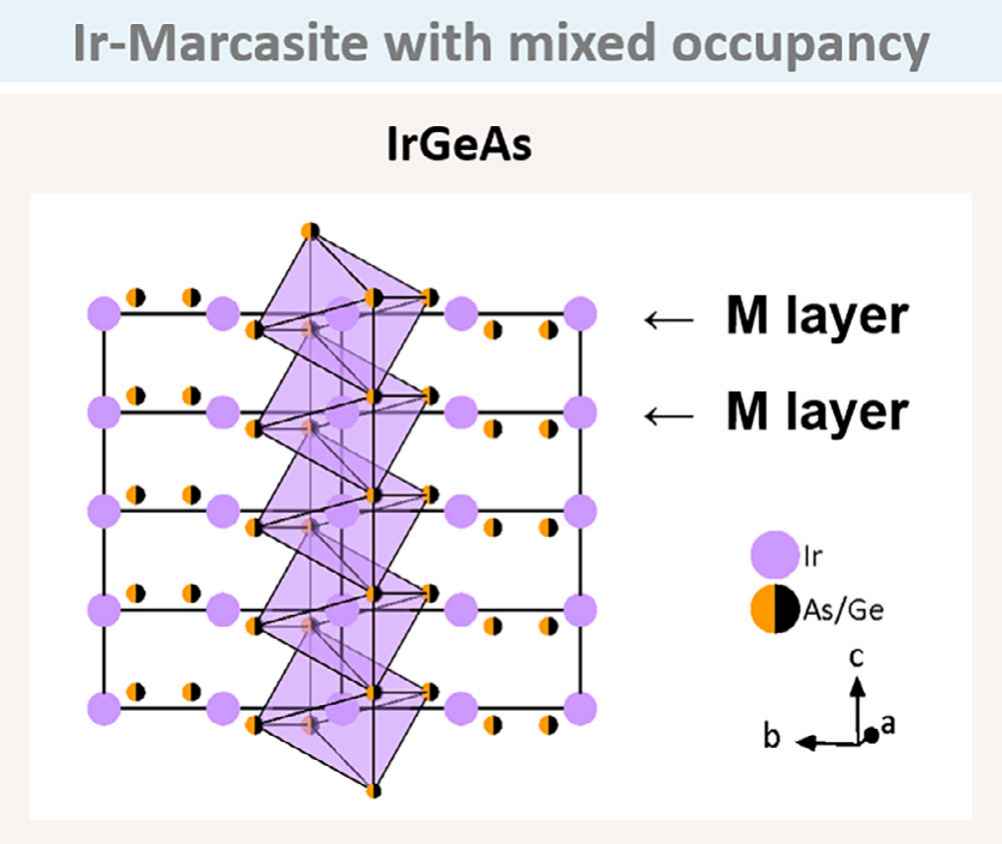
Crystallisation of IrGeAs in the marcasite structure type, with a mixed occupancy of Ge/As (half orange/black). The two marcasite layers within one unit cell are highlighted with arrows. The Ir atoms are equidistant along the octahedral chain. (Space group *Pnnm*).

The utility of the SDE rule becomes evident when considering the issue of Ge and As being essentially indistinguishable in X‐ray analysis. It is known that IrAs_2_ exhibits the arsenopyrite structure type, whereas the marcasite structure is not known for any Ir compound. With the SDE rule and the knowledge of Ir, Ge, and As, it can be predicted that the layer corresponding to the marcasite structure must be IrGeAs. To illustrate the concept, an attempt was made to synthesise the corresponding compound, which was successfully accomplished. As anticipated, the compound crystallised in the marcasite structure type (Figure 12). The occupancy of Ge and As was found to be mixed.

**Figure 13:**
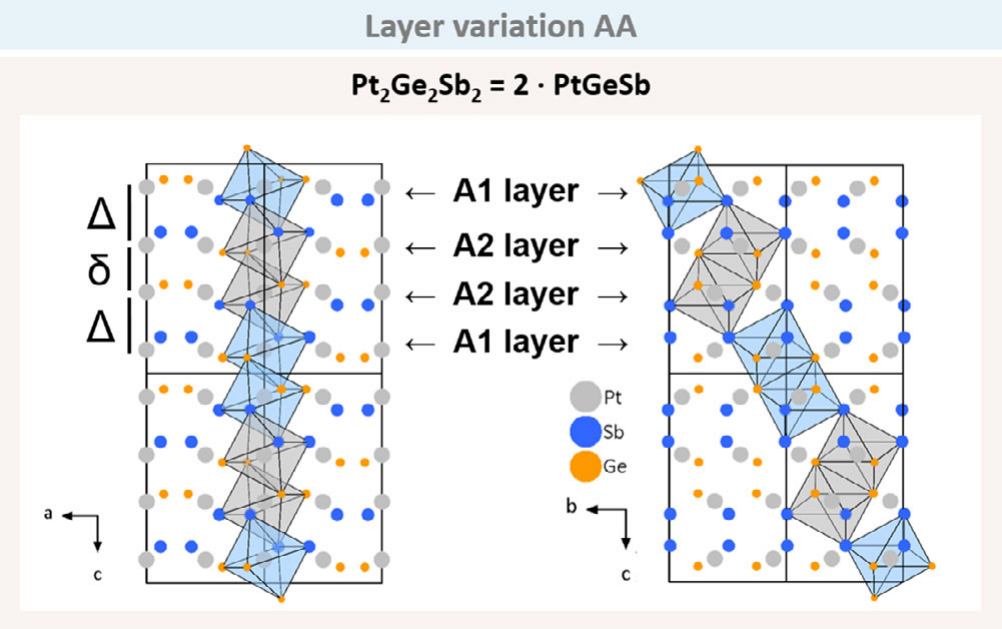
The *a‐c* and *b‐c* plane of PtGeSb crystallising in the ‘tilted double layer structure’ of arsenopyrite with layer A1 and A2 (AA, space group *Pbca*). The short (*δ*) and long (*Δ*) distances are shown on the left.

The Pt‐Ge‐Sb system exhibits a remarkable degree of diversity. The compound PtGeSb does not merely assume the arsenopyrite structure as illustrated above; it also forms a variant, which can be described as tilted double layers of arsenopyrite (AA) in the space group *Pbca* (Figure 13).

In the *a‐c* plane, the expected alternating pairs of Ge and Sb occur, consistent with the arsenopyrite structure type. However, the cell is doubled along the *c* axis compared to PtGeSb in the ‘basic’ arsenopyrite structure type. This is further elucidated in the *b‐c* plane, where the octahedra no longer form chains, but rather, the octahedra pairs are only corner‐sharing via the Sb atoms. This results in a rotation of approximately 60° and a doubled *c*‐axis. This structure type is named AA and correspondes to the structure of the mineral paracostibite (CoSbS).

**Figure 14:**
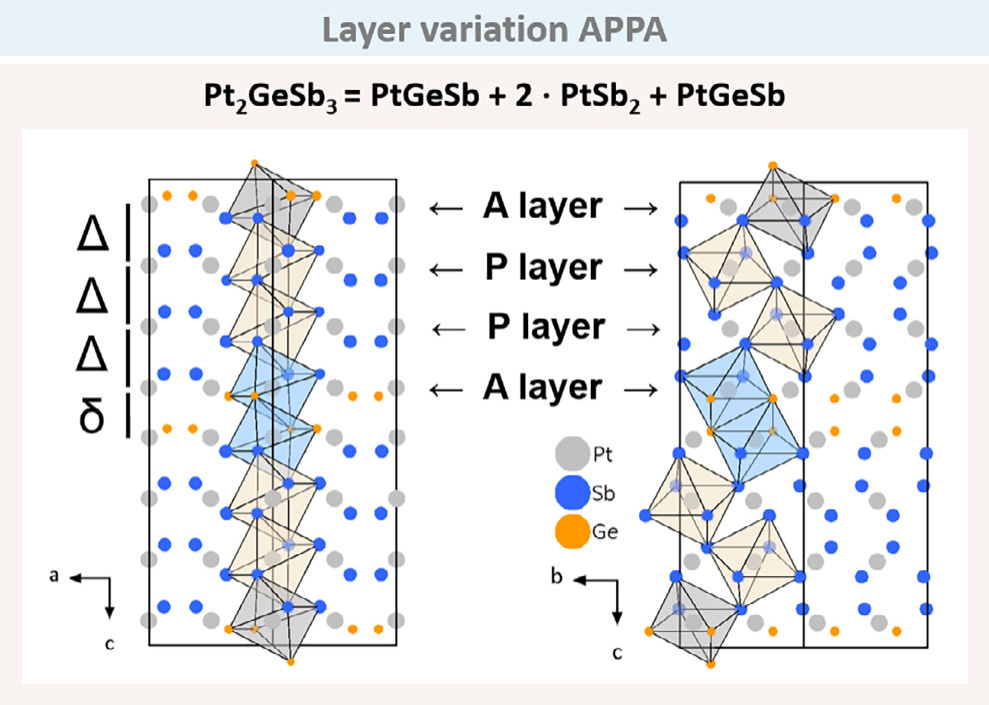
Pt_2_GeSb_3_ crystallising in the APPA structure variation (space group *Pbca*). The doubled cell is due to the rotation of the arsenopyrite layers, as highlighted by grey and blue octahedra. The respective layers are highlighted with arrows by A and P. The arrangement of the layers result in long (*Δ*) and short (*δ*) distances shown on the left.

A further variation, hitherto observed solely in Pt‐Ge‐Sb, is Pt_2_GeSb_3_, which also crystallises in the space group *Pbca*. The layers correspond to the structure layer variation APPA (Figure 14). This indicates the presence of a PtGeSb arsenopyrite layer (grey octahedra), followed by two PtSb_2_ pyrite layers (beige octahedra), and a final PtGeSb arsenopyrite layer (blue octahedra). The *c*‐axis is again doubled due to the rotation of the second arsenopyrite layer, as observed in the previous PtGeSb AA variation.

## Conclusion

The aim of our research was to understand and systematise compounds with the pyrite, marcasite or arsenopyrite structure types. For this purpose, we introduce an electron counting rule based on ′′SDE rule. In addition, we present a nomenclature for related compounds that exhibit layered variations of these structure types.

The SDE rule gives an easy way to predict and understand the three structure types marcasite, arsenopyrite, and pyrite based on the electrons not localised in single bonds. The idea is based on the distribution of electrons and the resulting more or less symmetrical spheres around the transition metal T.

$$\begin{array}{c} \text{SDE}=T_{\mathrm{VE}}-T_{t2g}+\left(X_{\mathrm{VE}}+Y_{\mathrm{VE}}\right)-8_{\sigma } \end{array}$$

(T_VE_: number of valence electrons (VE) of the transition metal. T_t2g_: number of electrons in the t_2g_ state for an octahedron (d_xy_, d_xz_ and d_yz_) with a maximum of six. X_VE_ and Y_VE_: VE of the main group element X and Y.) For all cases treated in this paper, we can simplify the SDE rule to have only two variables.

$$\begin{array}{cc} \text{SDE} & =T_{\mathrm{eg}}+X_{p}+Y_{p}-2\\ & =T_{\mathrm{eg}}+\text{Ep}-2 \end{array}$$

Where T_eg_ is the number of electrons in the e_g_ orbitals of the transition metal. X_p_ and Y_p_ are the number of electrons in the p orbital of the main group elements, combined to Ep.

The resulting number of electrons gives the corresponding structure type according to:

$$\begin{array}{cc} \text{SDE} & \leq 4\;\text{Marcasite}\\ \text{SDE} & =5\;\text{Arsenopyrite}\\ \text{SDE} & \geq 6\;\text{Pyrite} \end{array}$$

The electron rule agrees very well with existing compounds and allows the prediction of compounds with compositions, that would otherwise be hard to analyse. It can be used to identify the elements of a specific marcasite, arsenopyrite, or pyrite structure, or to predict the structure that a certain combination of elements will adopt. An example of this is Ir_3_GeAs_5_ where the second layer is found to be a marcasite type layer. With SDE = 4 this layer must contain Ge. This simplifies the X‐ray analysis drastically considering the weak X‐ray contrast of Ge and As. The other two layers are pyrite layers and must be IrAs_2_ layers according to SDE = 5. The combination of all three layers leads to the sum formula Ir_3_GeAs_5_, which could be confirmed by EDX measurements.

As a proof of concept, nine new ternary compounds were synthesised in the Pt/Ir‐Ge‐As/Sb systems. All structure models were calculated from single crystal X‐ray diffraction (Table S3) and all of them are combinations of marcasite, arsenopyrite, or pyrite structure type layers. Applying the SDE rule to each layer gives the corresponding structure type, abbreviated as M, A or P. In this way, each compound can be categorised based on the layers it contains. Ir_3_GeAs_5_, for example, can be called an AMA variant. This nomenclature is applied to all new compounds and their composition confirmed by EDX measurements (Table S2).

Additionally, the new compounds with different M, A or P layers feature, as far as we know for the first time, hetero‐ and homoatomic dumbbells within the same compound.

Unlike previous models introduced in the 1970s, the SDE rule is independent of formal charges and the fulfilment of the octet rule. The SDE rule not only explains and categorises all existing and newly discovered compounds, but also allows the prediction of other new compounds and possible variations.

The limitations of the model must also be emphasised. On the one hand, exceptions to the SDE rule seem to occur when the size difference between the participating elements becomes too big. To address this issue, we conducted a condensed analysis of 68 TX_2_ compounds from the ICSD database,^[^ ^49^, ^50^ ^]^ which resulted in a 95.6% agreement with the SDE rule. On the other hand, the model is based on empirical observations and is so far supported by the existing compounds. We do not claim that it allows quantitative findings on chemical bonding. Further investigations must show to what extent it proves itself. However, preliminary COHP investigations seem to support the concept.

A next step could be to analyse the influence of the different layers on the physical properties. Preliminary results show, for example, a strong influence of the layer type on the electrical conductivity. In addition, the bonding situation of the new compounds can be investigated by experiments, such as XPS or even ESR, confirming the so far promising concept of the SDE rule with respect to the electron distribution and independence of formal charges.

This work provides a new perspective on compounds that adopt the pyrite, marcasite, and arsenopyrite structure type and shows the much greater structural diversity and potential of layer variations than previously expected. The synthesis of many new compounds is possible, increasing our understanding of chemical bonding in compounds beyond the Zintl phases.

## Supporting information

The authors have cited additional references within the Supporting Information.^[^ ^51^, ^52^, ^53^, ^54^, ^55^, ^56^, ^57^, ^58^, ^59^, ^60^, ^61^, ^62^, ^63^, ^64^, ^65^, ^66^, ^67^, ^68^ ^]^

## Conflict of Interests

The authors declare no conflict of interest.

## Supporting information



Supporting Information

## Data Availability

The data that support the findings of this study are available from the corresponding author upon reasonable request.
