# Supplementary material for: Decoding Variants of Pyrite, Arsenopyrite, and Marcasite Using an Electron Counting Rule
Source: Angew Chem Int Ed Engl. 2025 May 5;64(23):e202502322. doi: 10.1002/anie.202502322 (PMC12124446; doi:10.1002/anie.202502322)
Supplement: Supplementary file 1 — Supporting Information [file ANIE-64-e202502322-s001.docx]

SUPPORTING INFORMATION

**Decoding Variants of Pyrite, Arsenopyrite and Marcasite Using an Electron Counting Rule**

Kristian Witthaut,^[a]^ Sandra Kreiner, ^[a]^ Dirk Johrendt*^[a]^

[a] Department of Chemistry, Ludwig-Maximilians-Universität München
Butenandtstrasse 5-13 (D), 81377 München, Germany
E-Mail: johrendt@lmu.de

**Table of Contents**

[Experimental Procedures 3](#_Toc192515659)

[Solid state synthesis and starting materials 3](#_Toc192515660)

[Scanning electron microscopy (SEM) and energy dispersive X-ray (EDX) spectroscopy 3](#_Toc192515661)

[Single crystal X-ray diffraction (sc-XRD) 3](#_Toc192515662)

[Powder X-ray diffraction (PXRD) and Rietveld refinement 3](#_Toc192515663)

[Density functional theory (DFT) calculations 3](#_Toc192515664)

[Results and Discussion 5](#_Toc192515665)

[EDX measurements 5](#_Toc192515666)

[Rietveld refinements 5](#_Toc192515667)

[Crystallographic information 6](#_Toc192515668)

[DFT calculations 6](#_Toc192515669)

[Agreement of the SDE rule with literature data 7](#_Toc192515670)

[References 10](#_Toc192515671)

Experimental Procedures

Solid state synthesis and starting materials

All compounds as well as the precursor PtGe were synthesised in a high-temperature solid-state reaction in resistance-heated, temperature regulated tube ovens with alumina crucibles sealed in silica vials under purified argon. Heating and cooling rates of 25 Kh^‑1^ were used. For PtGe stoichiometric amounts of elemental Pt (99.999 %, powder, Sigma Aldrich) and Ge (99.999 %, granules, Sigma Aldrich) were heated to 1173 K for a duration of 80 h. For all compounds the starting materials and specific reaction times are listed in Table S1. All compounds can be synthesised from stoichiometric mixtures of the elements Pt (99.999 %, powder, Sigma Aldrich), Ge (99.999 %, granules, Sigma Aldrich), As (99.99999+ %, chunks, Alfa Aesar) and Sb (99.9999 %, pellets, Alfa Aesar ) at a reaction temperature of 1273 K. In order to obtain single phase materials, the use of precursors seems to be necessary. This was tried exemplary for the compound Pt_2_GeSb_3_ with PtGe as precursor. All compounds are gray / black powders stable in both air and water.

**Table S1.** Overview of the elements and reaction times for the synthesis of the new compounds listed. A synthesis temperature of 1000°C with heating and cooling rates of 25 °Ch^-1^ were used.

| formula | PtGeAs | PtGeSb | IrGeAs | Pt_3_Ge_2_As_4_ | Pt_3_Ge_2_Sb_4_ | Pt_3_Ge_4_As_2_ | Ir_3_GeAs_5_ | PtGeSb | Pt_2_GeSb_3_ |
| --- | --- | --- | --- | --- | --- | --- | --- | --- | --- |
| space group | *P*2_1_/*c* (No. 14) | | *Pnnm* (No. 58) | *P*2_1_/*c* (No. 14) | | | | *Pbca* (No. 61) | |
| Pt / Ir (mg) | 173.64 | 144.20 | 169.71 | 170.80 | 150.26 | 106.76 | 139.05 | 126.64 | 16.02 |
| Ge (mg) | 81.90 | 120.00 | 64.14 | 63.60 | 55.95 | 79.50 | 52.55 | 94.31 | 43.98 (PtGe) |
| As / Sb (mg) | 44.46 | 35.80 | 66.15 | 65.60 | 93.79 | 41.00 | 108.40 | 79.05 | 40.00 |
| Reaction time | 80 h | 40 h | 50 h | 40 h | 80 h | 57 h | 80 h | 40 h | 80 h |

Scanning electron microscopy (SEM) and energy dispersive X-ray (EDX) spectroscopy

EDX spectra were measured using a Carl Zeiss EVO-MA 10 SEM with SE and BSE detectors and equipped with a Bruker Nano EDX detector (X-Flash detector 410-M). Data were collected and processed with the smartSEM^[1]^ and quantax 200^[2]^ software. Elements contained in the sample holder and adhesive carbon tabs were disregarded.

Single crystal X-ray diffraction (sc-XRD)

Single Crystal Data were collected with a D8 Quest diffractometer (Mo-Kα radiation, Göbel mirror optics, Photon-II CPAD detector). apex4^[3]^and sadabs^[4]^ were used for intensity integration and absorption correction. For this purpose suitable single crystals were selected from polycrystalline samples with Dual-Thickness MicroMounts (10 μm, MiTeGen, Ithaca, NY 14852, USA). The space group was chosen with XPREP^[5]^ based on systematic absences. The phase problem was solved with the Direct Methods (shelxt)^[6, 7]^ package and refinement of the structure was performed with the shelxl^[6]^ package. Structures were visualized using Diamond3^[8]^

Powder X-ray diffraction (PXRD) and Rietveld refinement

For PXRD measurements the samples were ground and sealed in glass capillaries (Hilgenberg GmbH, Marsfeld, Germany, 0.3 mm diameter). The capillaries were centered in a rotating goniometer head, data were subsequently collected on a STOE Stadi-P diffractometer (STOE & Cie GmbH, Darmstadt, Germany, CuKα_1_ radiation (*λ* = 1.540593 Å) or Ag-K*α*_1_ radiation (*λ =* 0.55942 Å), Ge(111) monochromator, Myhten 1k strip detector(Dectris, Baden, Switzerland)) in a modified Debye-Scherrer geometry via the software package winX^POW13 [9]^ and fitted with the MATCH!3 package^[10]^ based on single crystal data.

To demonstrate that phase purity can be achieved, a Rietveld refinement with the program GSASII was carried out.^[11]^ During the refinement process, lattice parameter, and fractional coordinates were freely refined. The peak profiles were described with the fundamental parameter approach. A potentially preferred orientation of the crystallites was accounted for with a 4^th^ order harmonic function and the background was modeled by a shifted Chebyshev polynomial.^[12, 13]^ The results were plotted using Origin.^[14]^

Density functional theory (DFT) calculations

First principle electronic structure calculations were performed based on density functional theory (DFT) and plane wave basis sets using the Vienna ab initio simulation package (VASP)^[15-17]^. Projector augmented waves (PAW)^[18, 19]^ were used and contributions of correlation and exchange were treated in the generalized-gradient approximation (GGA), as described by Perdew, Burke and Ernzerhof (PBE)^[20]^. The Brillouin zones were sampled with appropriate Г-centered *k*-point meshes with a resolution of at least 0.2/Å. The electronic energy convergence criterion was set to 10^‑4^ eV for the optimization and 10^-7^ eV for the subsequent static calculations.

Results and Discussion

EDX measurements

EDX measurements were carried out on at least five different crystallites of each compound. No other elements than Pt/Ir, Ge and As or Sb were detected. The results confirm all compositions as received from sc-XRD measurements.

**Table S2.** EDX data of all new compounds confirming the composition received from sc-XRD and the SDE rule. All ratios are shown in % and compared to the theoretical expected values. Standard deviations are given in round brackets.

| Formula (space group; Layer) | Pt / Ir (at.-%) | Ge (at.-%) | As / Sb (at.-%) |
| --- | --- | --- | --- |
| PtGeAs (*P*2_1_/*c*; A) calc. | 33 | 33 | 33 |
| PtGeAs (*P*2_1_/*c*; A) | 32.7(2) | 34.3(1) | 33.0(2) |
| Pt_3_Ge_4_As_2_ (*P*2_1_/*c*; AMA) calc. | 33 | 44 | 22 |
| Pt_3_Ge_4_As_2_ (*P*2_1_/*c*; AMA) | 32(5) | 45.7(8) | 22.2(8) |
| Pt_3_Ge_2_As_4_ (*P*2_1_/*c*; APA) calc. | 33 | 22 | 44 |
| Pt_3_Ge_2_As_4_ (*P*2_1_/*c*; APA) | 36.6(5) | 18.9(9) | 44.5(9) |
| PtGeSb (*P*2_1_/*c*; A) calc. | 33 | 33 | 33 |
| PtGeSb (*P*2_1_/*c*; A) | 31.2(4) | 32.7(3) | 36.1(4) |
| PtGeSb (*Pbca*; AA) calc. | 33 | 33 | 33 |
| PtGeSb (*Pbca*; AA) | 31.8(3) | 32.2(5) | 36.0(5) |
| Pt_3_Ge_2_Sb_4_ (*P*2_1_/*c*; APA) calc. | 33 | 22 | 44 |
| Pt_3_Ge_2_Sb_4_ (*P*2_1_/*c*; APA) | 34.6(3) | 21.3(3) | 44.1(3) |
| Pt_2_GeSb_3_ (*Pbca*; APPA) calc. | 33 | 16 | 50 |
| Pt_2_GeSb_3_ (*Pbca*; APPA) | 30.6(3) | 19.2(4) | 50.0(7) |
| IrGeAs (*Pnnm*; M) calc. | 33 | 33 | 33 |
| IrGeAs (*Pnnm*; M) | 32.9(4) | 32.2(5) | 30.9(4) |
| Ir_3_GeAs_5_ (*P*2_1_/*c*; AMA) calc. | 33 | 11 | 56 |
| Ir_3_GeAs_5_ (*P*2_1_/*c*; AMA) | 35.6(4) | 11.7(1) | 52.8(4) |

Rietveld refinements

Rietveld refinements for both IrGeAs and Pt_2_GeSb_3_ were performed. For all compounds containing Pt the binaries PtAs_2_ and PtSb_2_ are formed. Using PtGe as a precursor proved to be promising to avoid both side phases. Figure S1 shows the phase pure sample of IrGeAs and Figure S2 shows the sample of Pt_2_GeSb_3_ with a fraction above 90 %.


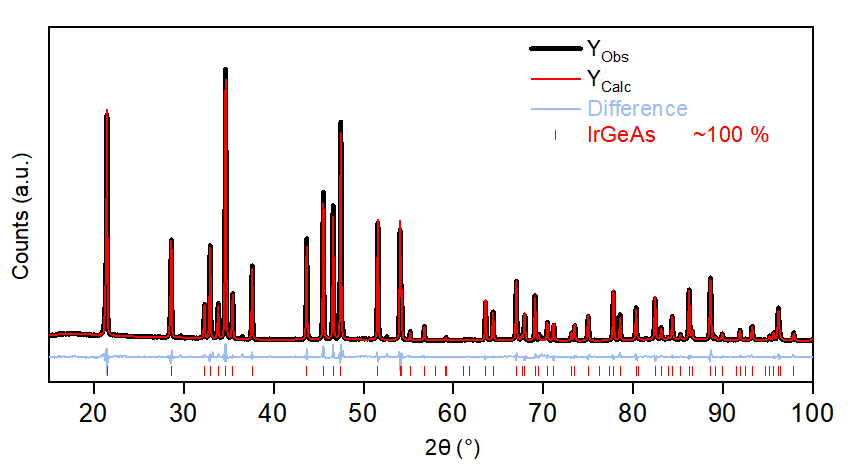


**Figure S1.** Result of the Rietveld refinement of IrGeAs. Observed intensities (Y_Obs_, black line, CuKα_1_, *λ* = 1.540593 Å), calculated intensities (Y_Calc_, red line) and difference plot (gray line). Positions of Bragg reflections of IrGeAs are given as red bars.


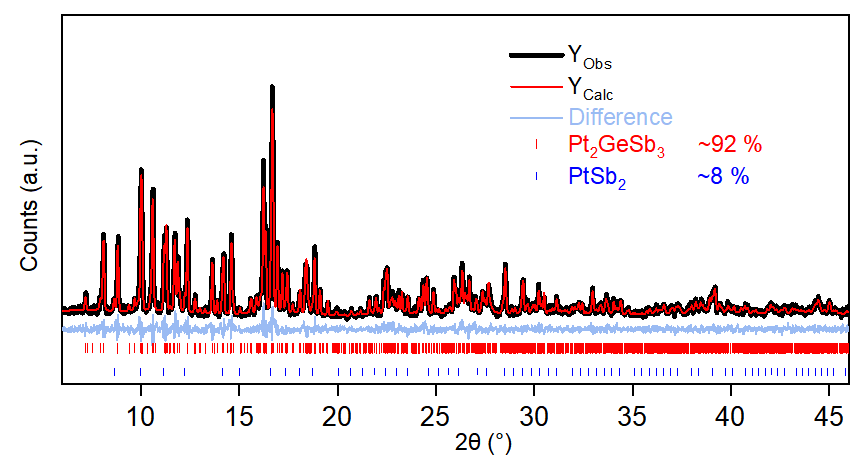


**Figure S2.** Result of the Rietveld refinement of Pt_2_GeSb_3_. Observed intensities (Y_Obs_, black line, AgKα_1_, *λ =* 0.55942 Å), calculated intensities (Y_Calc_, red line) and difference plot (gray line). Positions of Bragg reflections of IrGeAs and PtSb_2_ are given as red bars or blue bars, respectively.

Crystallographic information

**Table S3:** Summary of the crystallography data of all new compounds in the systems of Ir/Pt-Ge-As/Sb. All standard deviations are given in round brackets.

| formula | PtGeAs | PtGeSb | IrGeAs | Pt_3_Ge_2_As_4_ | Pt_3_Ge_2_Sb_4_ | Pt_3_Ge_4_As_2_ | Ir_3_GeAs_5_ | PtGeSb | Pt_2_GeSb_3_ |
| --- | --- | --- | --- | --- | --- | --- | --- | --- | --- |
| deposition number | 2400935 | 2400934 | 2400932 | 2400931 | 2400936 | 2400929 | 2400930 | 2400933 | 2400937 |
| space group | *P*2_1_/*c* (No. 14) | | *Pnnm* (No. 58) | *P*2_1_/*c* (No. 14) | | | | *Pbca* (No. 61) | |
| layer variation | A | | M | APA | | AMA | | AA | APPA |
| *a* / Å | 6.1744(9) | 6.3424(6) | 5.5568(4) | 8.5196(3) | 8.9511(12) | 8.299(2) | 8.2158(3) | 6.4147(2) | 6.4141(2) |
| *b* / Å | 6.2434(9) | 6.3940(6) | 6.2312(4) | 6.1750(2) | 6.4468(7) | 6.229(1) | 6.1408(2) | 6.4488(2) | 6.4385(2) |
| *c* / Å | 6.2325(10) | 6.3693(6) | 2.9198(2) | 6.1649(2) | 6.4432(9) | 6.321(1) | 6.2008(2) | 11.4756(3) | 24.2308(7) |
| *β* / ° | 115.479(7) | 115.658(5) | - | 92.990(2) | 92.573(7) | 100.20(3) | 97.438(2) | - | - |
| *V*_cell_ / Å^3^ | 216.89(6) | 232.83(4) | 101.10(1) | 323.89(2) | 371.44(8) | 321.56(1) | 310.21(2) | 474.71(2) | 1000.8(1) |
| Z | 4 | | 2 | 2 | 2 | 2 | 2 | 8 | 8 |
| *ρ*_X-ray_ / g cm^-3^ | 10.492 | 10.658 | 11.159 | 10.563 | 10.635 | 10.591 | 10.961 | 10.898 | 10.991 |
| *μ /* mm^-1^ | 92.950 | 82.361 | 96.341 | 93.885 | 77.569 | 93.519 | 95.278 | 82.239 | 77.410 |
| *R*_σ_ / *R*_int_ (BASF) | 0.0420 / 0.0606 | 0.0489 / 0.0708 | 0.0274 / 0.0478 | 0.0221 / 0.0356 | 0.0287 (0.2291) | 0.0409 / 0.0524 | 0.0261 / 0.0422 | 0.0276 / 0.0586 | 0.0223 / 0.0506 |
| *R*_1_ (F > 4σ(F)) / all | 0.0365 / 0.0389 | 0.0304 / 0.0442 | 0.0157 / 0.0200 | 0.0216 / 0.0267 | 0.0374 / 0.0421 | 0.0283 / 0.0379 | 0.0179 / 0.0237 | 0.0240 / 0.0292 | 0.0451 / 0.0472 |
| *wR*_2_ (F^2^ > 2σ(F^2^)) / all | 0.0946 / 0.0983 | 0.0545 / 0.0592 | 0.0384 / 0.0405 | 0.0417 / 0.0432 | 0.0972 / 0.1011 | 0.0645 / 0.0678 | 0.0351 / 0.0369 | 0.0450 / 0.0468 | 0.0815 / 0.0818 |
| GooF | 1.123 | 1.093 | 1.173 | 1.164 | 1.127 | 1.116 | 1.069 | 1.153 | 2.654 |
| Δ*ρ*_max/min_ / eÅ^-3^ | 3.073 /  ‑ 3.461 | 2.565 /  ‑ 2.672 | 1.671 /  - 1.572 | 1.480 / - 1.559 | 4.915 / - 2.808 | 2.869 / - 2.172 | 1.353 / - 1.685 | 1.818 / - 1.755 | 3.223 / - 3.782 |

The deposition numbers 2400929-2400937 (detailed in Table S3) contain the crystallographic data for this paper. These data are provided free of charge by the joint Cambridge Crystallographic Data Centre and Fachinformationszentrum Karlsruhe Access Structures service [www.ccdc.cam.ac.uk/structures](http://www.ccdc.cam.ac.uk/structures)

DFT calculations

DFT calculations were performed to support the assignment of Ge and As as obtained from the SDE rule. The first approach was to calculate the formation energies of the three introduced Pt-Ge-As compounds by subtracting the energies of the elements (*Materials Project* database^[21]^). The resulting energies were normalised to eV atom^-1^ and converted to kJ mol^-1^ for better comparison. Next, all Ge and As positions were swapped and the energies of the “inverse” structures were calculated analogously.

The result is visualised in Figure S3 and allows some conclusions. Pt_3_Ge_2_As_4_ is the most stable of the three variants with an energy of ‑52.421 kJ mol^-1^. PtGeAs has a slightly higher energy of ‑50.332 kJ mol^‑1^. Pt_3_Ge_4_As_2_ has a significantly higher energy of ‑44.597 kJ mol^-1^, but remains below the energy of both of the inverted structures “Pt_3_Ge_4_As_2_” (‑42.227 kJ mol^‑1^) and “Pt_3_Ge_2_As_4_” (‑41.215 kJ mol^-1^). Especially the difference between the original Pt_3_Ge_2_As_4_ and the inverted “Pt_3_Ge_4_As_2_” with -10.194 kJ mol^-1^ is remarkably high. The trend in the energy of the original structures seems to show a destabilisation of the compounds with increasing Ge ratio. This is also consistent with the great difficulty in obtaining the pure phase of Pt_3_Ge_4_As_2_.


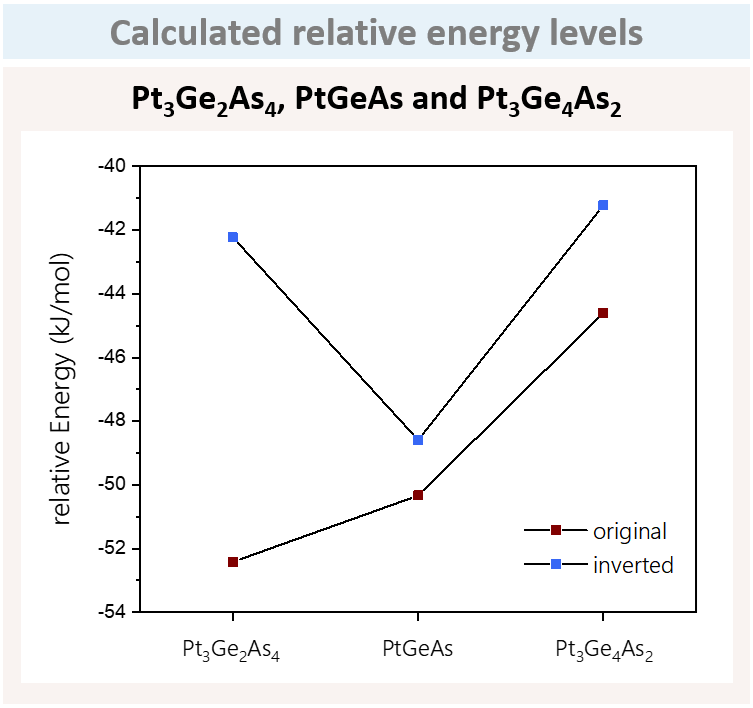


**Figure S3:** The DFT energy levels for Pt_3_Ge_2_As_4_, PtGeAs and Pt_3_Ge_4_As_2_ show that the assignment of Ge and As from the SDE-rule is unambiguously favoured over the inverted model with interchanged Ge and As atoms.

A more general understanding was obtained by investigating the influence of Ge and As on the distance of Pt-Pt between two neighbouring octahedra (Figure S4a). The distances Pt-Ge-Pt (red) and Pt-As-Pt (blue) were varied from 2.8 to 3.9 Å in 14 steps for three Pt(Ge_3_As_3_) edge-sharing octahedra (Figure S4b). This structure cut-out was optimised in each step while keeping the Pt-Pt distances fixed. The result is very unambiguous and agrees very well with the observed data. In PtGeAs the experimental Pt-Ge-Pt distance is 2.95 Å and the Pt-As-Pt distance is 3.64 Å. These values are marked in Figure 16a, as red and blue lines, respectively. Focusing on the calculated values for Pt-Ge-Pt in red shows that the minimum energy is very close to the found Pt-Ge-Pt distance of about 3 Å, while it increases rapidly with increasing distance. The opposite is observed for the Pt-As-Pt distance in blue, where the minimum energy is again found close to the experimental one around 3.6 Å and a strong increase in energy is observed approaching 3 Å.


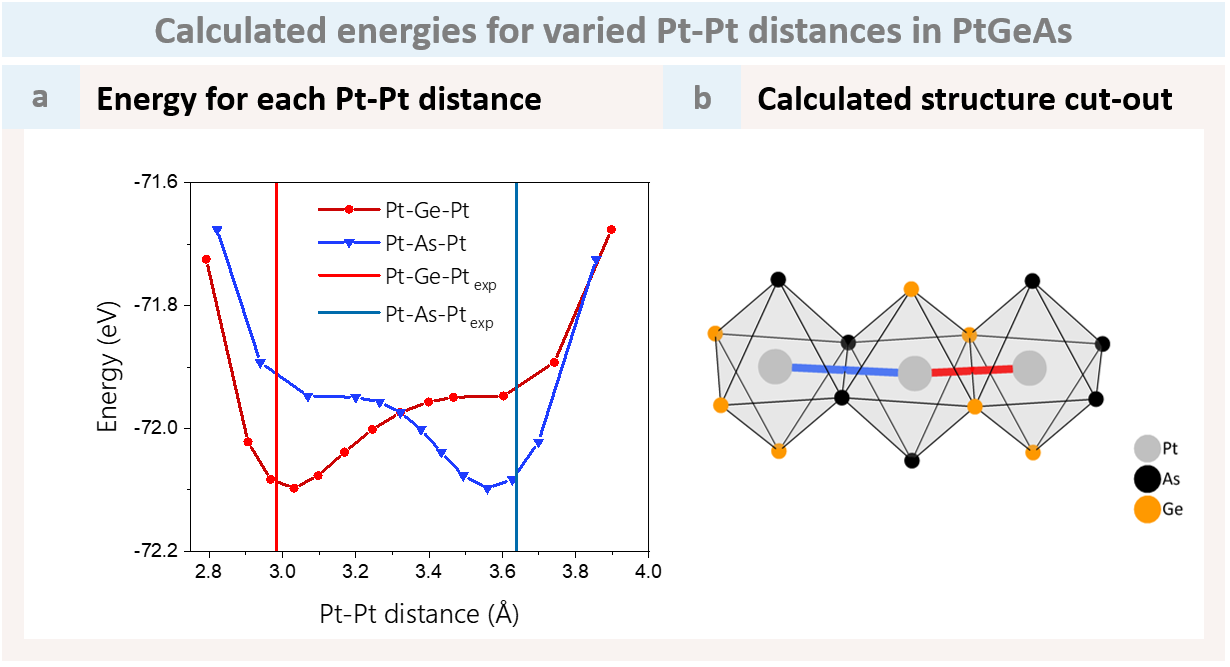


**Figure S4:** a) The energy minimum for both the Pt-As-Pt (blue) and Pt-Ge-Pt (red) distances reach their minimum close to the experimental Pt-Pt distances (blue and red vertical line), when assigning Ge and As according to the SDE-rule. The energy increases significantly when approaching the opposite Pt-Pt distance (e.g. crossover between Pt-Ge-Pt energy and the Pt-As-Pt experimental distance). b) The calculated structure cut-out from PtGeAs (P2_1_/c). The Pt-As-Pt distance is highlighted in blue and the Pt-Ge-Pt distance is highlighted in red.

Agreement of the SDE rule with literature data

To assess the overall agreement of the SDE rule with known literature data, we analysed compounds from the ICSD Database.^[22, 23]^ Due to processing constraints, we restricted our analysis to a specific subset of compounds. We focused on transition metals (T) from groups 4 (Cr group) to 12 (Zn group) and main group elements (X) from group 3 (B group) to 6 (O group). Additionally, we only considered binary compounds with the stoichiometry TX2, within the structure types of Marcasite, Arsenopyrite, and Pyrite. This approach excluded ternary compounds and those with related structure types, such as Ullmannite or Cobaltite. After removing duplicate entries based on chemical composition, we were left with 68 compounds. These are listed in Table S4, along with their respective space group, transition metal (T), its number of d electrons (T_d_), the number of electrons in the e_g_ orbitals (T_eq_), and the main group element (X) with its number of p electrons (X_p_). The value from the SDE rule is calculated by SDE = T_eg_ + 2 ∙ X_p_ – 2.

The last two columns display the predictions from the SDE rule and the corresponding experimental structure type. Agreement is shown in green and by ✓, disagreement is shown marked in red.

The table indicates that, out of the 68 compounds, only 3 have been incorrectly assigned to Pyrite rather than Marcasite. This means that the SDE rule agrees with the experimental data in 95.6% of the cases.

**Table S4:** Summary for the agreement of the SDE rule with compounds from the ICSD Database^[22, 23]^. For all TX_2_ compounds the respective space group, the transition metal (T), with its number of d electrons (T_d_) and electrons in the e_g_ orbitals (T_eq_), as well as the main group element (X) with its number of p electrons (X_p_) are shown. The SDE column shows the calculated SDE value and the agreement with the experimental structure type in green by ✓, or disagreement marked in red.

| Space group | T | T_d_ | T_eg_ | X | Xp | SDE | | Experimental Structure Type |
| --- | --- | --- | --- | --- | --- | --- | --- | --- |
| *P* 1 2_1_/*c* 1 | Co | 7 | 1 | P | 3 | 5 | ✓ | Arsenopyrite |
| *P* 1 2_1_/*c* 1 | Co | 7 | 1 | Sb | 3 | 5 | ✓ | Arsenopyrite |
| *P* 1 2_1_/*c* 1 | Co | 7 | 1 | As | 3 | 5 | ✓ | Arsenopyrite |
| *P* 1 2_1_/*c* 1 | Rh | 7 | 1 | P | 3 | 5 | ✓ | Arsenopyrite |
| *P* 1 2_1_/*c* 1 | Ir | 7 | 1 | P | 3 | 5 | ✓ | Arsenopyrite |
| *P* 1 2_1_/*c* 1 | Ir | 7 | 1 | Bi | 3 | 5 | ✓ | Arsenopyrite |
| *P* 1 2_1_/*c* 1 | Ir | 7 | 1 | As | 3 | 5 | ✓ | Arsenopyrite |
| *P* 1 2_1_/*c* 1 | Rh | 7 | 1 | As | 3 | 5 | ✓ | Arsenopyrite |
| *P* 1 2_1_/*c* 1 | Ir | 7 | 1 | Sb | 3 | 5 | ✓ | Arsenopyrite |
| *P* 1 2_1_/*c* 1 | Rh | 7 | 1 | Sb | 3 | 5 | ✓ | Arsenopyrite |
| *P* 1 2_1_/*c* 1 | Rh | 7 | 1 | Bi | 3 | 5 | ✓ | Arsenopyrite |
| *P* 1 2_1_/*c* 1 | Ir | 7 | 1 | Bi | 3 | 5 | ✓ | Arsenopyrite |
| *P* 1 2_1_/*c* 1 | Rh | 7 | 1 | Bi | 3 | 5 | ✓ | Arsenopyrite |
| *P* 1 2_1_/*c* 1 | Pd | 8 | 2 | Br | 5 | 10 | ✓ | Pyrite |
| *P a* $\bar{3}$ | Fe | 6 | 0 | S | 4 | 6 | ✓ | Pyrite |
| *P a* $\bar{3}$ | Mn | 5 | 0 | Te | 4 | 6 | ✓ | Pyrite |
| *P a* $\bar{3}$ | Mn | 5 | 0 | S | 4 | 6 | ✓ | Pyrite |
| *P a* $\bar{3}$ | Mn | 5 | 0 | Se | 4 | 6 | ✓ | Pyrite |
| *P a* $\bar{3}$ | Pt | 8 | 2 | P | 3 | 6 | ✓ | Pyrite |
| *P a* $\bar{3}$ | Ni | 8 | 2 | P | 3 | 6 | ✓ | Pyrite |
| *P a* $\bar{3}$ | Pt | 8 | 2 | As | 3 | 6 | ✓ | Pyrite |
| *P a* $\bar{3}$ | Pd | 8 | 2 | Sb | 3 | 6 | ✓ | Pyrite |
| *P a* $\bar{3}$ | Zn | 10 | 4 | O | 4 | 10 | ✓ | Pyrite |
| *P a* $\bar{3}$ | Ru | 6 | 0 | Te | 4 | 6 | ✓ | Pyrite |
| *P a* $\bar{3}$ | Ru | 6 | 0 | S | 4 | 6 | ✓ | Pyrite |
| *P a* $\bar{3}$ | Ru | 6 | 0 | Se | 4 | 6 | ✓ | Pyrite |
| *P a* $\bar{3}$ | Pd | 8 | 2 | As | 3 | 6 | ✓ | Pyrite |
| *P a* $\bar{3}$ | Pt | 8 | 2 | Bi | 3 | 6 | ✓ | Pyrite |
| *P a* $\bar{3}$ | Co | 7 | 1 | S | 4 | 7 | ✓ | Pyrite |
| *P a* $\bar{3}$ | Ni | 8 | 2 | S | 4 | 8 | ✓ | Pyrite |
| *P a* $\bar{3}$ | Cu | 9 | 3 | S | 4 | 9 | ✓ | Pyrite |
| *P a* $\bar{3}$ | Os | 6 | 0 | S | 4 | 6 | ✓ | Pyrite |
| *P a* $\bar{3}$ | Os | 6 | 0 | Te | 4 | 6 | ✓ | Pyrite |
| *P a* $\bar{3}$ | Rh | 7 | 1 | Te | 4 | 7 | ✓ | Pyrite |
| *P a* $\bar{3}$ | Os | 6 | 0 | Se | 4 | 6 | ✓ | Pyrite |
| *P a* $\bar{3}$ | Cd | 10 | 4 | O | 4 | 10 | ✓ | Pyrite |
| *P a* $\bar{3}$ | Au | 9 | 3 | Sb | 3 | 7 | ✓ | Pyrite |
| *P a* $\bar{3}$ | Co | 7 | 1 | Se | 4 | 7 | ✓ | Pyrite |
| *P a* $\bar{3}$ | Pt | 8 | 2 | Sb | 3 | 6 | ✓ | Pyrite |
| *P a* $\bar{3}$ | Rh | 7 | 1 | Se | 4 | 7 | ✓ | Pyrite |
| *P a* $\bar{3}$ | Rh | 7 | 1 | S | 4 | 7 | ✓ | Pyrite |
| *P a* $\bar{3}$ | Cd | 10 | 4 | S | 4 | 10 | ✓ | Pyrite |
| *P a* $\bar{3}$ | Cd | 10 | 4 | Se | 4 | 10 | ✓ | Pyrite |
| *P a* $\bar{3}$ | Cu | 9 | 3 | Te | 4 | 9 | ✓ | Pyrite |
| *P a* $\bar{3}$ | Ir | 7 | 1 | S | 4 | 7 | ✓ | Pyrite |
| *P a* $\bar{3}$ | Rh | 7 | 1 | Te | 4 | 7 | ✓ | Pyrite |
| *P a* $\bar{3}$ | Ru | 6 | 0 | S | 4 | 6 | ✓ | Pyrite |
| *P a* $\bar{3}$ | Ru | 6 | 0 | Se | 4 | 6 | ✓ | Pyrite |
| *P a* $\bar{3}$ | Ru | 6 | 0 | Te | 4 | 6 | ✓ | Pyrite |
| *P a* $\bar{3}$ | Zn | 10 | 4 | S | 4 | 10 | ✓ | Pyrite |
| *P a* $\bar{3}$ | Zn | 10 | 4 | Se | 4 | 10 | ✓ | Pyrite |
| *P a* $\bar{3}$ | Fe | 6 | 0 | S | 4 | 6 | ✓ | Pyrite |
| *P a* $\bar{3}$ | Ni | 8 | 2 | S | 4 | 8 | ✓ | Pyrite |
| *P n n m* | Pt | 8 | 2 | O | 4 | 8 | Pyrite | Marcasite |
| *P n n m* | Pt | 8 | 2 | Ge | 2 | 4 | ✓ | Marcasite |
| *P n n m* | Pt | 8 | 2 | Ge | 2 | 4 | ✓ | Marcasite |
| *P n n m* | Fe | 6 | 0 | P | 3 | 4 | ✓ | Marcasite |
| *P n n m* | Ru | 6 | 0 | As | 3 | 4 | ✓ | Marcasite |
| *P n n m* | Cr | 4 | 0 | Sb | 3 | 4 | ✓ | Marcasite |
| *P n n m* | Fe | 6 | 0 | As | 3 | 4 | ✓ | Marcasite |
| *P n n m* | Ru | 6 | 0 | P | 3 | 4 | ✓ | Marcasite |
| *P n n m* | Ru | 6 | 0 | Sb | 3 | 4 | ✓ | Marcasite |
| *P n n m* | Os | 6 | 0 | P | 3 | 4 | ✓ | Marcasite |
| *P n n m* | Os | 6 | 0 | As | 3 | 4 | ✓ | Marcasite |
| *P n n m* | Os | 6 | 0 | Sb | 3 | 4 | ✓ | Marcasite |
| *P n n m* | Ni | 8 | 2 | Sb | 3 | 6 | Pyrite | Marcasite |
| *P n n m* | Cu | 9 | 3 | Se | 4 | 9 | Pyrite | Marcasite |

# References

[1] *SmartSEM*, Version 5.07 Beta, Carl Zeiss Microscopy Ltd.: Cambridge, UK

[2] *QUANTAX 200*, Version 1.9.4.3448, Bruker Nano GmbH: Berlin, Germany, **2013**.

[3] *APEX4*, v2021.10-0, Bruker AXS Inc.: Madison, USA, **2021**.

[4] G. M. Sheldrick, *SADABS: Multi-Scan Absorption Correction*, Version 2016/2, Bruker AXS Inc.: Madison, WI, USA, **2012**.

[5] G. M. Sheldrick, *XPREP*, Version 2008/2, Bruker AXS Inc.: Madison, USA, **2008**.

[6] G. M. Sheldrick, *SHELXL-2018/3, Acta Crystallogr., Sect. C: Struct. Chem. ,* **2018**, 71 (1), 3–8.

[7] G. M. Sheldrick, *SHELXT 2018/2: A programm for crystal strcutre solution*, University of Göttingen: Germany, **2018**.

[8] K. Brandenburg, Diamond Ver. 3.2k, **2014**.

[9] *WinXPow: STOE Powder Software*, Version 3.0.2.5. ed., STOE & Cie GmbH: Darmstadt, Germany, **2011**.

[10] H. Putz, *MATCH!3*, Version: 3.9.0.158, Crystal Impact: Bonn, Germany, **2019**.

[11] B. H. Toby, R. B. Von Dreele, *J. Appl. Cryst.,* **2013**, 46, 544–549.

[12] R. W. Cheary, A. A. Coelho, J. P. Cline, *J. Res. Natl. Instl. Stand. Technol.,* **2004**, 109, 1–25.

[13] R. W. Cheary, A. Coelho, *J. Appl. Crystallogr.,* **1992**, 25, 109–121.

[14] OriginPro 2019B OriginLab Corporation, Northhampton (USA), **1991−2019**.

[15] G. Kresse, J. Furthmoeller, *Comput. Mat. Sci.,* **1996**, 6 (1), 15–50.

[16] G. Kresse, J. Hafner, *Phys. Rev. B* **1994**, 49 (20), 14251–14269.

[17] G. Kresse, J. Furthmoeller, *Phys. Rev. B,* **1996**, 54.

[18] G. Kresse, D. Joubert, *Phys. Rev.,* **1999**, 59.

[19] P. E. Blöchl, *Phys. Rev. B,* **1994**, 50 (24), 17953–17979.

[20] J. Perdew, K. Burke, M. Ernzerhof, *Phys. Rev. Lett.,* **1996**, 77 (18), 3865–3868.

[21] A. Jain, S. P. Ong, G. Hautier, W. Chen, W. D. Richards, S. Dacek, S. Cholia, D. Gunter, D. Skinner, G. Ceder, K. A. Persson, *APL Mater,* **2013**, 1.

[22] NIST Standard Reference Database Number 3 NIST Inorganic Crystal Structure Database, *ICSD*, 20899, National Institute of Standards and Technology: Gaithersburg MD, DOI: https://doi.org/10.18434/M32147, **02.2024**.

[23] I. D. Brown G. Bergerhoff, *Crystallographic Databases*, F.H. Allen et al. (Hrsg.) Chester: International Union of Crystallography, **1987**.
